# Draft Genome Sequence of Multidrug-Resistant *Mycobacterium tuberculosis* Clinical Isolate OSDD515, Belonging to the Uganda I Genotype

**DOI:** 10.1128/genomeA.00750-13

**Published:** 2013-11-21

**Authors:** Shamsudheen Karuthedath Vellarikkal, Ajay Vir Singh, Pravin Kumar Singh, Parul Garg, Viswa Mohan Katoch, Kiran Katoch, Open Source Drug Discovery Consortium, D. S. Chauhan, Sridhar Sivasubbu, Vinod Scaria

**Affiliations:** Genomics and Molecular Medicine, CSIR Institute of Genomics and Integrative Biology, Delhi, Indiaa; GN Ramachandran Knowledge Center for Genome Informatics, CSIR Institute of Genomics and Integrative Biology, Delhi, Indiab; National JALMA Institute of Leprosy and other Mycobacterial Diseases, Tajganj, Agra, Indiac; CSIR Open Source Drug Discovery Unit, Anusandhan Bhavan, New Delhi, Indiad

## Abstract

We describe the genome sequencing and analysis of a clinical isolate of the multidrug-resistant *Mycobacterium tuberculosis* Uganda I genotype (OSDD515) from India.

## GENOME ANNOUNCEMENT

*Mycobacterium tuberculosis* is a fastidious pathogen and etiological agent of tuberculosis, a major cause of mortality and morbidity, causing >1.4 million deaths in 2010 alone (1). A number of major and minor genotypes have been reported from across the world, of which the major lineages include the East Asian (Beijing), East African-Indian, Indo-Oceanic, Euro-American (Haarlem, LAM, T, and X), West African I, and West African II lineages. The complete or draft genome sequences of many of the members of the major lineages are now available in the public domain (2, 3), and they show significant differences in their gene repertoires and sometimes in their genome architectures. It is now imperative to understand the genome structures and gene repertoires of the minor genotypes, which have been less explored. The Uganda 1 genotype was first identified in the Kampala region of Uganda, and it is prevalent in East Africa (4). This genotype has been reported to have a higher infectious rate and lower drug resistance than other genotypes. The genotype is majorly restricted to eastern and central Africa but has also been reported in other regions of the world (5–7).

In this paper, we describe the draft genome of a clinical isolate of *M. tuberculosis*, confirming its clustering in the Uganda I family spoligotype. The clinical isolate *M. tuberculosis* OSDD515 was obtained from the strain repository maintained at the National JALMA Institute for Leprosy and other Mycobacterial Diseases and is part of the Open Source Drug Discovery Open Access Repository. Spoligotyping, a drug sensitivity test, and DNA isolation were performed as per the standard protocols. The drug sensitivity test showed that OSDD515 is resistant to streptomycin, rifampin, isoniazid, ethambutol, cycloserine, ethionamide, capreomycin, and pyrazinamide and is sensitive to amikacin, ofloxacin, kanamycin, and *para*-aminosalicylate sodium. The raw sequence data were generated after library preparation on an Ion Torrent PGM platform as per the protocols recommended by the manufacturers. Draft genomes were assembled *de novo* using the CLC Genomics Workbench 6.0.5. The assembly resulted in 127 contigs with an N_50_ value of 60,965 bp and a total assembly of 4,206,725 bp. Further, the automated gene prediction of the draft genomes was performed using the RAST server (8). The analysis revealed 4,175 genes, including 48 RNA genes.

### Nucleotide sequence accession numbers.

This whole-genome shotgun project has been deposited at DDBJ/EMBL/GenBank under the accession no. AUXC00000000. The version described in this paper is version AUXC01000000.
